# Give a Man a Fish or Teach a Man to Fish: A Cross-Level Moderated Mediation Model of Cognitive and Performance Responses of Team Members to Help of Team Leaders

**DOI:** 10.3389/fpsyg.2021.618834

**Published:** 2021-07-23

**Authors:** Yanting Zhu, Lihua Zhang, Yungui Guo

**Affiliations:** ^1^School of Labor and Human Resources, Renmin University of China, Beijing, China; ^2^School of Business, Hunan University of Science and Technology, Xiangtan, China

**Keywords:** autonomy-oriented help, dependency-oriented help, leader-member exchange, self-efficacy, individual work role performance, cross-level interaction

## Abstract

Drawing upon theoretical lenses of social cognitive theory, this study explores whether, when, why, and how the helping behaviors of team leaders influence individual work role performance of team members (in terms of individual task proficiency, task adaptivity, and task proactivity) through self-efficacy of team members. The consequences of different types of help of leaders are uncovered in this study. By proposing a cross-level moderated mediation model and using multisource and multistage data from 303 team members in 39 work teams, autonomy-oriented help of leaders was found to have a differential effect on individual work role performance of members *via* the self-efficacy of the latter when controlling for dependency-oriented help of leaders. Moreover, the multilevel analysis of moderation uncovered that leader–member exchange relationship at the team level engendered a boundary condition for the mediating role of member self-efficacy in the relationship between autonomy-oriented help of leaders and individual work role performance of members in this model.

## Introduction

Helping behavior, as “an important form of citizenship behavior” (Podsakoff et al., 2000, p. 516), has gained great attention in academia. It is referred to as spontaneously helping others with work-related problems (Podsakoff et al., 2000) and can happen every day through interactions between coworkers (e.g., Alvarez and van Leeuwen, 2011; Koopman et al., 2016) in work teams. However, previous research has focused on help seeking (e.g., Nadler, 1997; Bamberger, 2009; Komissarouk et al., 2017), feedback seeking (Ashford et al., 2003), and help providing (e.g., Flynn, 2006; Maki et al., 2017), but lacked to explore the psychological mechanism of receiving help in depth (for exceptions, see Alvarez and van Leeuwen, 2011, 2015; Halabi et al., 2011; Alvarez et al., 2018).

In work teams, a team leader is responsible for coordination within the team (Zaccaro et al., 2001), helping members with problem solving by providing informational and advisory guidance (Courtright et al., 1989), and ensuring the services delivery of the team and collective goal achievement. In this way, the help and support of team leaders can generate significant impacts on the perceptions and behaviors of team members (Li et al., 2015). However, findings on the consequences of obtaining leader support are inconsistent for employees. Some studies have examined that leader support can promote several forms of proactive employee behaviors (e.g., Ohly et al., 2006; Parker and Wu, 2014); while others have reported an insignificant relationship between them (e.g., Frese et al., 1999). Further, scholars have appealed that it is necessary to study various types (other than “a simple act,” Fisher et al., 2018, p. 1525) of helping behaviors, and investigate the psychological process (such as “who, what, when, why, and how”) of specific contents or types of help of leaders on employees' behaviors (Ehrhart, 2018, p. 2; Wu and Parker, 2017). To better address such inconsistency and explore different working mechanisms of receiving help from team leaders, this study explores three aspects: different types of help behavior, the mediating process of help of leaders (why help of leaders is important, how it contributes to employee work performance, whether the effects of different types of help of leaders are differential), and boundary condition (when help of leaders matters).

As the old saying goes, “give a man a fish and you feed him for a day; teach a man to fish and you feed him for a lifetime.” Apparently, it involves different types of help and suggests an “either/or” helping solution in life. In the helping literature, researchers have made substantial efforts in the categorization of helping behavior, such as autonomy-oriented help and dependency-oriented help (Nadler, 2002) according to individual needs, necessary help and convenient help (Gross and McMullen, 1983), instrumental help (task-oriented and informational) and emotional help (personal but less task-oriented; Bamberger, 2009), job-related and non-job-related support (Bowling et al., 2005), task assistance and social and emotional support (Mor Barak et al., 2009), reactive helping (other-oriented and prosocial) and proactive helping (helper self-interested; Spitzmuller and Van Dyne, 2013), as well as four types of helping from the perspective of the cost to the helper including causal helping (low cost), substantial personal helping (high cost), emotional helping, and emergency helping (due to close relationships; McGuire, 1994). However, few studies have integrated the categorization of helping behaviors with leader or supervisory support, or have compared the differential effects of different types of leader helping between each other in a work team setting. In so doing, we first investigate two types of helping behaviors of leaders, which are in line with individual needs of employees (Nadler, 1997): autonomy-oriented and dependency-oriented help. Due to their differential impacts on competence, self-esteem, and resource gaining of individuals, these two types of help have received great attention in academia (e.g., Nadler, 1997, 2002; Alvarez and van Leeuwen, 2011, 2015; Alvarez et al., 2018). Specifically, autonomy-oriented help involves “providing the recipients with the tools to solve their problems on own” (Nadler, 2002, p. 491); while dependency-oriented help is short-term information or benefits provided to recipients who will need similar support from help givers again when confronting similar issues (Nadler, 2002; Nadler and Chernyak-Hai, 2014).

Second, we drew on social cognitive theory (Bandura, 1986) and adopted a member-centric approach to uncover *how, why*, and *whether* receiving different types of helping behaviors of team leaders might differently affect work performance of team members. In so doing, we propose and examine how different types of helping behaviors of team leaders can generate differential effects on work role performance of team members *via* their sense of self-efficacy. The social cognitive theory posits that self-efficacy (“the belief in one's capabilities to organize and execute the courses of action required to produce given attainments;” Bandura, 1997, p. 3) is the central mechanism of human agency (Bandura, 1997), which can shed light on how the relationship plays out between help of team leaders and work performance of team members. In a team environment, team members with a robust sense of self-efficacy can generate confidence in task execution, boost “intrinsic interest and deep engrossment” in task behaviors, set challenging work goals, adapt to changes or uncertainty, and make persistent efforts toward goal accomplishments (Bandura, 1994, p. 2), which, in turn, facilitate their work performance.

Third, we further explored *when* receiving different types of help of leaders facilitates self-efficacy of team members and, in turn, their work performance. In Chinese society, the quality of leader–member relationship is a critical contextual factor to employee behavior and performance (Yu et al., 2016). The common use of work teams and teamwork in organizations has increasingly stressed the importance of considering team-level stimuli, such as leadership climate on the motivational process of team members (Chen and Kanfer, 2006). Thus, beyond perceiving leader–member exchange (LMX) at the individual level, group-level LMX can be positively associated with leadership climate and team atmosphere (Yang and Tan, 2016), as well as can influence employee work performance especially for Chinese workers who may still work hard for their leaders even if being treated not so well (Rockstuhl et al., 2012). Leveraging the social cognitive theory, we propose that group-level LMX can interact with receiving help of team leaders to influence the improvement of self-efficacy of team members. We also propose that the indirect effect of member self-efficacy is contingent upon the factor of group-level LMX at a cross-level framework.

This study will make four main contributions as follows; first, this study will contribute to the helping literature by improving the understanding of autonomy- and dependency-oriented help and enriching research findings on the consequences of receiving these two types of helping behaviors. Prior research has not yet discovered their effects on employee work performance. Second, it will uncover why, how, and when processes of these two types of helping behaviors of team leaders, and further compare their differential effects. Up to date, few literature has opened this black box. Third, it will contribute to the group-level LMX theory by examining the boundary condition of group-level LMX in the theoretical framework in this research. Few studies have built up a multi-level perspective to explore the potential effects of LMX at the group level. Fourth, this study will also enhance the understanding of antecedents of work role performance, which few studies have examined whether and which certain leader behaviors can facilitate employee work role performance. Finally, this research will further deepen the understanding and applications of the social cognitive theory from a multilevel research framework.

## Theory and Hypotheses Development

### Help of Team Leaders, Self-Efficacy of Team Members, and Their Work Role Performance

As previously stated, some studies have supported the positive role of leader support in enhancing employee performance. The uncertainty and dynamics of the modern work environment urge organizations to establish more requirements for individual work performance of employees (Griffin et al., 2007). However, despite being equipped with sufficient knowledge, skills, abilities, or other resources such as the help of leaders, people make decisions to take actions and perform based on their own sense of self-efficacy (Bandura, 1997). They choose to act when they believe they can (Bandura, 1997). Such global evaluation and judgment about one's own capabilities drive individuals to unfold their agency for work performance (e.g., a total set of roles and responsibilities; see Griffin et al., 2007). Research has theoretically and empirically shown that self-efficacy can trigger positive consequences, such as proactive work behaviors (Wu and Parker, 2017), employee creativity (Liao et al., 2010; Yu et al., 2016), and positive objective performance change (Liu et al., 2017). It is a critical cognitive-motivational construct by impacting “individual choices, goals, emotional reactions, efforts, coping, and persistence” (Gist and Mitchell, 1992, p. 198). Drawing upon the social cognitive theory, a widely recognized theory used to understand human behaviors in a social context (Smith and Hitt, 2005) and the multilevel phenomenon of the impact of leader behavior on perceptions of subordinates (e.g., Liao et al., 2010), self-efficacy is posited as one of the strongest psychological drivers inherent in individual behaviors in various environments (Bandura, 1994, 1997). With such a strong theoretical base, we propose that generally in a team setting, the help behaviors of team leaders may impact the self-efficacy of team members, which, in turn, influence member work role performance. Specifically, we also expect that the potential differential effects of different types of leader helping exist during this process.

#### Different Types of Help and Similar Constructs

Extensive studies have been conducted on supervisory helping behavior (Hu et al., 2018). The constructs of autonomy-oriented help and dependency-oriented help of leaders show a certain resemblance to perceived supervisory support (PSS). PSS is defined as “the degree to which supervisor value [employees'] contributions and care about their well-being” (Eisenberger et al., 2002, p. 565; Kottke and Sharafinski, 1988). It is regarded as general views or perceptions of subordinates on receiving support from their leader (e.g., Eisenberger et al., 2002; Edmondson and Boyer, 2013; Wang et al., 2014; Chen et al., 2016; Yang et al., 2018). Providing guidance, arranging good assignments and flexible work schedules, and showing consideration are typical examples of perceived supervisory support (Maertz et al., 2007; Yang et al., 2018). However, these are not content-specific forms of leader support. Wu and Parker (2017) proposed a specific type of leader secure-base support (in three form of availability, encouragement of growth, and non-interference) given the different content of leader support may influence the proactivity of an employee in different ways. Consistent with the research of Wu and Parker (2017), this study focuses on the help of team leaders with different contents: autonomy-oriented help and dependency-oriented help. These two constructs are distinguishable from PSS and other similar ones regarding general leader support.

Specifying different types of help can better address more issues in the helping process within organizations (Ehrhart, 2018). From the perspectives of the individual need for autonomy, autonomy-oriented help is identified as a practice in which the help giver provides tools to the help recipient to solve problems, such as similar problems in the future, which is of great educational value (Nadler, 1997, 2002; Nadler and Chernyak-Hai, 2014). The practice has been found to be more self-supporting and self-competent with positive feelings (Alvarez and van Leeuwen, 2011), and positively correlated to empowerment, decision making, and beliefs in personal or family change (Alvarez et al., 2018). In contrast, dependency-oriented help is referred to as helping others by providing a full solution for problem-solving (Nadler, 1997, 2002). This form of help offers high instrumentality to help recipients, so they can solve urgent problems immediately (Alvarez and van Leeuwen, 2011).

#### Different Types of Help and Self-Efficacy

Bandura (1982) pinpointed a hierarchy of four major and effective sources of self-efficacy in broad areas: (1) mastery experience, an individual's direct experience, and previous successful performance, which is the most direct and influential factor in self-efficacy enhancement; (2) vicarious experience, or the observational learning experiences gained from role models or similar referents; (3) verbal persuasion, in which individuals are verbally persuaded to be able to master certain tasks; and (4) physiological states, which can be referred as the somatic and emotional states that can influence people's assessments of their capabilities. These four sources are not mutually exclusive but interdependent. Mastery experience forms a direct and stable source of self-efficacy; whereas the other three (modeling, verbal persuasion, emotion arousal) are relatively indirect sources. Besides internal determinants of self-efficacy (such as knowledge, skills, abilities, motivation, effort, and performance strategies, which are under personal control), external factors, such as interdependence, resources, and the environment of tasks, as well as the interpersonal environment in an organization, can also impact an individual's assessment of their capabilities of performing work tasks (Gist and Mitchell, 1992). Hence, the help of a team leader may develop the self-efficacy of team members mainly *via* these four cues proposed by Bandura (1982). We expect that both autonomy-oriented help and dependency-oriented help of team leaders can contribute to the self-efficacy of team members. Specifically, the autonomy-oriented help of a team leader may foster member self-efficacy through enactive mastery, social persuasion, and positive physiological states. And the dependency-oriented help of leaders may enhance member self-efficacy through vicarious experience, verbal instructions, and secure physiological states. Therefore, the two different types of leader helping can accentuate the level of member self-efficacy in different ways.

##### Autonomy-Oriented Help and Self-Efficacy

Previous studies have stressed the positive consequences of autonomy-oriented help for help recipients. For instance, autonomy-oriented help can improve the sense of self-competence, empowerment, social support, and autonomy of a help receiver (Nadler, 1997, 2002; Alvarez and van Leeuwen, 2011, 2015). The autonomy-oriented help of a team leader can positively change the self-efficacy of their team members in both cognitive and motivational ways. With autonomy orientation, the team leader helps their subordinates learn how to use tools (e.g., knowledge, skills, critical thinking strategies, performance strategies) to analyze and solve problems. In support of the model of self-efficacy determinants proposed by Gist and Mitchell (1992), this leads to a gain in internal self-efficacy sources of team members from abilities (with low variability) to performance strategies (analytical, psychological, behavioral strategies, with high variability). Individual abilities typically act as internal and stable determinants of self-efficacy, which can satisfy a team member's psychological need for autonomy (Alvarez and van Leeuwen, 2011) and self-reliance (Nadler and Halabi, 2015). In addition, performance strategies as the highest variable and internal cues may easily contribute to boosting a positive change in self-efficacy, since they are easy for a person to control. For team members, this can increase the possibility of their repeated successful performance, mastery experience, and, in turn, raise the perceptions of self-efficacy at work (Bandura, 1997; Parker, 1998).

Team members' beliefs in their capabilities can also be strengthened through verbal persuasion by the leader (Bandura, 1997). For example, when providing autonomy-oriented help, a team leader is more likely to express confidence in specific capabilities of subordinates, offer autonomy to them, and verbally persuade and encourage them to take over the task by using the tools to solve problems on their own. Team members are more likely to believe that they are capable (of performing this task) because the leader thinks they can do it. Autonomy-oriented help of team leaders is also often regarded as leaders granting valuable information, resources, and training in core competencies to team members, which makes the latter feel positive at work. Such positive physiological and affective states bolster the self-efficacy development of team members (Bandura, 1997). Furthermore, when receiving autonomy-oriented help, team members may indirectly gain self-efficacy *via* their vicarious experience (Bandura, 1997). For example, the subordinate earns an opportunity to observe how the team leader unfolds and analyzes problems, and to reflect on how to adapt previous knowledge to current difficulties. Therefore, the autonomy-oriented help of a team leader can be positively associated with the self-efficacy of team members.

##### Dependency-Oriented Help and Self-Efficacy

The extant research on receiving dependency-oriented help has stressed the negative consequences for help receivers, such as the risk of showing inferiority, threat on positive social identity (Nadler, 2002), and lack of independence and resources (Alvarez and van Leeuwen, 2015) in a team. Scholars found that help givers had the propensity to provide dependency-oriented help over autonomy-oriented help to maintain their advantages of status and territory of expertise, as well as to control the dependence of help seekers (Nadler, 2002; van Leeuwen and T?uber, 2010). Dependency-oriented help implies status inequality between similar individuals (Nadler, 1997, 2002; van Leeuwen et al., 2011). However, a high status of the help giver can offset negative impacts on the feelings of the help receiver (Alvarez and van Leeuwen, 2011). For example, if the help giver is an expert or represents authority other than being a peer of the help receiver, the help receiver may not perceive status incongruence or negative feelings when receiving dependency-oriented help. In this case, it can be anticipated that receiving dependency-oriented help from a team leader would not trigger feelings of inferiority compared with receiving help from coworkers (Alvarez and van Leeuwen, 2011). In work teams, team leaders have several reasons to offer dependency-oriented help, depending on the level of member competency (Nadler, 2002), the urgency of business demands (Alvarez and van Leeuwen, 2015), or self-protection purpose of a leader (Nadler, 2002). Despite such varying motivations, we contend that the instrumental helpfulness of the dependency-oriented help of a team leader can positively affect member self-efficacy through the two determinants of self-efficacy: vicarious experience and physiological states.

Particularly, the dependency-oriented help of a team leader can be more directly effective in an urgent business situation than autonomy-oriented help because of its instrumental nature for performers experiencing low self-efficacy in challenging work assignments. Bandura (1997) argued that modeling is effective to promote the self-efficacy of an individual. When a leader offers dependency-oriented help, they provide a full and detailed solution to a problem (Nadler, 1997), such as step-by-step guidance and operating procedures. Such direct informational inputs bridge a channel of modeling for team members. Specifically, when problems are brought to the team leader, the leader asks members for more information to generate a full solution or detailed verbal instructions. Such process acts as a vicarious experience for the subordinates, in which they can observe how the leader comes up with the elaborate approach; further, they can deduce basic performance strategies from the provided solution and apply them to similar issues next time (although they may still need certain support from leaders). In some occasions, team members have already had a solution in mind but they are afraid of trying and failure. These team members can, thus, benefit from the dependency-oriented help of the leader and achieve a sense of self-efficacy through self-verification during the process of being helped. Dependency-oriented help can provide team members with a sense of security and reduce their fear, worries, and anxiety about tackling “no-clue” issues. Moreover, it can indirectly improve the possibility of successful direct performance of team members, especially for low performers. Although those team members may not know the rationale behind the scenario and could not master skills to solve a similar problem on their own next time, the dependency-oriented help of leaders at least allows them to apply the same solution for the next try, and aids them in “paying help forward” (Alvarez and van Leeuwen, 2015, p. 1). To a certain extent, such work experiences can increase self-efficacy perceptions of team members.

Scholars have argued that people prefer receiving autonomy-oriented help than dependency-oriented help for three main reasons. First, autonomy-oriented help, which drives an individual's growth in competence (e.g., knowledge, skills, abilities), is self-enhancing, empowering, and provides the help receiver with greater need satisfaction, as well as cultivates a closer help giver-and-receiver relationship (Nadler, 1997, 2002; Weinstein and Ryan, 2010; van Leeuwen et al., 2011). Second, it can make a more positive impression within the group compared with receiving dependency-oriented help (van Leeuwen and T?uber, 2010). Third, receiving autonomy-oriented help creates more positive feelings than dependency-oriented help, since autonomy-oriented helper shows respect for the need for autonomy of a recipient (Alvarez and van Leeuwen, 2015). Aside from these factors, people tend to feel more comfortable receiving autonomy-oriented help from “high-status” individuals with authority (e.g., expert, supervisor, manager) than from their peers (Alvarez and van Leeuwen, 2011). By integrating the helping literature and the self-efficacy theory in the work team context, both types of help of leaders can serve as drivers of perceived self-efficacy of team members at work. However, their consequences can be different. Compared with dependency-oriented help, autonomy-oriented help of team leaders can promote the self-efficacy of team members more by contributing to internal determinants (e.g., abilities), direct personal attainments, and other indirect sources. Thus, we propose the following:

Hypothesis 1: Compared with dependency-oriented help, team leader autonomy-oriented help is more positively related to team member self-efficacy.

### Self-Efficacy and Individual Work Role Performance

In previous research, individual efficacy has been found to contribute to motivation and performance (Bandura and Locke, 2003), such as academia performance (Chemers et al., 2001), job performance (Stajkovic and Luthans, 1998; Judge and Bono, 2001) and other forms of performance in diverse environments (e.g., school, workplace, hospital, and sports teams). It is highlighted that self-efficacy can play a pivotal role in an individual's interpretation of their work performance. For instance, Liao et al. (2010) demonstrated the connecting role of member self-efficacy between their interpretation of relationship quality and member creativity. Liu et al. (2017) empirically illustrated the mediating role of self-efficacy change in objective performance change through the interpretation of citizenship behaviors from the perspective of social exchange.

In light of social cognitive theory, personal self-efficacy is the foundation of human agency and the key psychological mechanism of individual performance in the work environment (Gist and Mitchell, 1992; Bandura, 1997, 2001). People make attempts at their work tasks only with the support of their propositional sense of self-efficacy (Bandura, 1997). Based on Bandura (2001), there are four essential characteristics of human agency: (1) intentionality with centering on the planning of actions (e.g., goal setting); (2) forethought, by anticipating the consequences of different actions and selecting actions to produce desired outcomes; (3) self-reactiveness, through self-regulation of motivation, affect, and action toward desired goals; and (4) self-reflectiveness, by reflecting the adequacy of one's own capabilities, thoughts, and actions. Thus, the perceived self-efficacy of team members can influence their cognitive, motivational, affective, and selection process to leverage skills, capabilities, and resourcefulness for work performance accomplishments (Bandura, 1994). Specifically, as planners, fore thinkers, self-regulators, and self-verifiers, team members can intentionally set more challenging work tasks and goals, regulate their motivation and affect to implement action plans for desired outcomes, and reflect to evaluate whether they have adaptive mindsets and adequate capabilities for actions at work.

In today's rapidly changing environment, organizations expect more of employee work performance on the adaptability and proactivity besides traditional job task fulfillment (e.g., proficiency). The highly dynamic work environment and changing nature of work in organizations have urged a need to adopt a more overarching way to assess employee work performance. Reflecting such interdependence and uncertainty of the business world, Griffin et al. (2007) proposed an integrated model of employee work role performance, which encompasses three dimensions of work role behaviors: task proficiency, task adaptivity, and task proactivity at three levels (individual, team, and organization). In this research, we focus on the effect of self-efficacy of team members on their individual work role performance in terms of individual task proficiency, adaptivity, and proactivity. Specifically, individual task proficiency involves those behaviors “that can be formalized and are not embedded in a social context,” which reflect “the degree to which an employee meets the known expectations and requirements of his or her roles as an individual” (Griffin et al., p. 331). It is closely associated with the expected performance of their own tasks, and it is “fundamentally about the required and expected types of individual performance” (Carpini et al., 2017, p. 547). Besides, individual task adaptivity and task proactivity are also necessarily included to evaluate individual work behaviors about adapting to dynamic environment needs and responding to uncertain changes. Individual task adaptivity is referred to as “the degree to which individuals cope with, respond to, and/or support changes that affect their roles as individuals” (Griffin et al., 2007, p. 331). Individual task proactivity is defined as “the extent to which individuals engage in self-starting, future-oriented behavior to change their individual work situations, their individual work roles, and themselves” (Griffin et al., 2007, p. 332).

When a team member experiences high self-efficacy, they tend to be more motivated in goal setting and put more effort and persistence toward achieving their goals (Bandura, 1997) to meet their role expectations and requirements. Moreover, they can self-acquire and learn new skills to better adapt to changes when feeling competent (Carpini et al., 2017). Furthermore, being high in the perception of self-efficacy of a subordinate can also drive subordinates themselves to better anticipate the likelihood of proactive actions to reach more desired outcomes, regulate their own motivation and affect, and have more confidence in their capabilities of proactivity, such as conducting new work procedures and proactive problem solving (Griffin et al., 2007). Thus, considering the relationships between these two types of leader helping and member self-efficacy, we propose the following:

Hypothesis 2a: Team member self-efficacy mediates the relationship between team leader autonomy-oriented help and individual work role performance of team members.

Hypothesis 2b: Team member self-efficacy mediates the relationship between team leader dependency-oriented help and individual work role performance of team members.

### The Cross-Level Moderating Effect of Leader–Member Exchange

Based on the LMX theory, at the dyad level, a team leader develops different relationships with his or her team members (Liden and Graen, 1980) given the limited time, resources, and energy of the leader (Graen and Uhl-Bien, 1995). Prior research has indicated that LMX was a significant contextual factor influencing employee attitudes (Duchon et al., 1986) and performance (Gerstner and Day, 1997), as well as in how followers interpret leader actions (e.g., Fisk and Friesen, 2012). Mature LMX can be perceived as an important resource channel for employee self-efficacy enhancement (Liao et al., 2010). In the eyes of team members, the team leader has more objective, essential but scarce job information, which is normally considered as a type of valuable job resource (Wang and Zhong, 2011). At the individual level, a low quality of LMX represents that the team member is the out-group person of his or her team leader, which the exchange relationship is limited to formal job descriptions and employment contract. In contrast, a high quality of LMX suggests that the team member is the in-group person of his or her team leader, which the leader–member relationship enjoys a high level of tangible (e.g., economic support) and intangible resources (e.g., mutual trust, well communications, respect, social support, mutual learning, mutual adaption, affection, and other intangible obligations) exchanges between them (Liden et al., 1993; Graen and Uhl-Bien, 1995), especially in the Chinese work context.

Although LMX was originated at the individually dyad level, researchers have appealed to be cautious that LMX effects exist across different levels (i.e., Schriesheim et al., 2001; Omilion-Hodges and Baker, 2013; Zhao et al., 2014). Meanwhile, dynamic needs from external and internal environments drive the common use of teams in organizations (Wang, 2020). In other words, we should not neglect the potential contextualizing effect of LMX at the team level (group-level LMX), which reflects the central tendency and overall level of relationship quality between leaders and members of the team (Liden et al., 2006). Especially, most employees in the Chinese society with a high level of collectivism tend to focus more on how leaders treat “us” well and, thus, are more likely to be influenced by group-level LMX (e.g., Yang, 2020). However, the importance of group-level LMX has not yet been fully paid attention (Yang and Tan, 2016). In the extant literature, the group-level LMX has been shown to be positively related to team potency (Boies and Howell, 2006), team affective commitment (Wang and Sun, 2013), team innovation (Li and Cheng, 2013), team performance (Wang and Sun, 2013), and team information elaboration (Zhao et al., 2014), as well as negatively related to team conflict (Boies and Howell, 2006). Moreover, the group-level LMX has been shown to play a moderating role in the relationship between group-level LMX differentiation and team productivity (Liden et al., 2006; Le Blanc and González-Romá, 2012). Specifically, when the group-level LMX is high, the group-level LMX differentiation would not impact team performance and affective commitment. However, when the group-level LMX is low, the group-level LMX differentiation can be positively associated with team performance and affective commitment. Meanwhile, Stewart and Johnson (2009) found that the group-level LMX can moderate the relationship between the interaction of team diversity and group-level LMX and team performance; as well as the relationship between team diversity and turnover rate. Besides its influences on team outcomes, the group-level LMX can also act as a contextual stimulus to affect the individual psychological processes of employees. For example, Yu et al. (2016) found that the group-level LMX can positively moderate the relationship between individual LMX and employee creative role identification, since group-level LMX can help foster a positive team atmosphere of leader empowerment, support, and interpersonal cooperation.

Apparently, LMX can be viewed as a valuable organizational resource (Omilion-Hodges and Baker, 2013). In a team with high group-level LMX, there is a tendency that leaders and their team members develop good, deep, and intensive exchange relationships with mutual trust, respect, obligations, resource exchange, and more empowerment (Boies and Howell, 2006; Ford and Seers, 2006). Their leaders form “trust, affect, and respect-based relationships” with them, and is more inclusive of them (Bauer and Erdogan, 2016, p. 3), as well as tend to have sufficient resources and present their willingness to share collective resources within their teams (Nishii and Mayer, 2009), and team members are more inclined to form collective identity and more willing to pay attention to the collective work tasks and goals other than personal goals (Omilion-Hodges and Baker, 2013). This can easily form a positive team climate of mutual respect and interpersonal cooperation (Cogliser and Schriesheim, 2000). Such resource distribution can also affect individual perceptions of justice and resource sharing between peers within a team (Omilion-Hodges and Baker, 2013). In turn, team members are inclined to feel more sense of support, autonomy, and empowerment to fulfill their challenging tasks within the team (Wang and Sun, 2013). Previous studies have indicated that the group-level LMX was positively related to member self-efficacy through the leader provision of developmental chances and positive verbal persuasion (Boies and Howell, 2006). In this case, the group-level LMX quality may engender potential cross-level effects on the relationship between leader helping and member self-efficacy. Specifically, when receiving the autonomy-oriented help of a leader in a team with high group-level LMX, the subordinates feel empowered and are more inclined to recognize the help of their leader as core and valuable resources (e.g., key information and knowledge) because of a high level of trust and respect in such positive team climate. It is also possible for them to receive instrumental resources, verbal encouragement, and other support accompanied with the autonomy-oriented help of their leader (Boies and Howell, 2006). On the other hand, team members tend to perceive more obligations within the team (Settoon et al., 1996; Wang and Sun, 2013) and be more committed to meeting expectations of the leader by acquiring new skills, new strategies, and conducting new ways of thinking to make good use of the autonomy-oriented help of the leader as a form of reciprocity based on the social exchange theory. Consequently, high group-level LMX can accentuate the positive relationship between the autonomy-oriented help of leaders and the self-efficacy of members. In contrast, the effect of the autonomy-oriented help of a leader on member self-efficacy would be weakened if the group-level LMX is low, since the autonomy-oriented help of leaders may not be interpreted as key resources in such teams, and team members are often lacking in developmental opportunities, leader empowerment, valuable resources, and other support. Hence, we propose the following:

Hypothesis 3a: Group-level LMX quality positively moderates the relationship between team leader autonomy-oriented help and team member self-efficacy.

In teams with high group-level LMX, team members can feel a high level of abundant resources from team leaders (Boies and Howell, 2006), and they tend to form nice interactions among each other in such a positive team climate, which encourages mutual trust and cooperation (Wang and Sun, 2013). Thus, the effect of the dependency-oriented help of leaders on the self-efficacy of members would be attenuated, since the functionality of sufficient developmental chances, cooperation of team members, and team-level resource sharing would substitute for the effect of the dependency-oriented help of leaders on the self-efficacy of members. On the contrary, in teams with low group-level LMX, leaders and their team members have much less mutual communication and fewer relational intangible exchanges (Tu and Lu, 2016). Team members tend to simply complete required work responsibilities and receive basic tangible compensation (Graen and Uhl-Bien, 1995). If receiving dependency-oriented help from the leader who directly involves in the problem schema, analyzes, and approaches a step-by-step solution, team members can receive intangible benefits in the form of informational knowledge and earn a good opportunity of observational learning which can raise their efficacious beliefs (Parker, 1998). In other words, in the context of low group-level LMX, team members may regard dependency-oriented help of leaders as additionally scarce resources (such as more information on performing tasks) and important vicarious experience beyond their transactional exchanges to form a higher level of self-efficacy. Consequently, the low group-level LMX may strengthen the positive relationship between dependency-oriented help and self-efficacy. Hence, we propose the following:

Hypothesis 3b: Group-level LMX quality negatively moderates the relationship between team leader dependency-oriented help and team member self-efficacy.

To step forward, we anticipate that team members in a team who experience high group-level LMX perceive a better fit between their expectations and the actual receipt of the autonomy-oriented help of a team leader, which triggers a higher sense of self-efficacy and in turn higher level of work role performance by socially interpreting such help as gaining more support, trust, and unique resources from their leader, and having more opportunities for successful mastery experiences. In contrast, in work teams with low group-level LMX, team members tend to value the dependency-oriented help of leaders as intangible benefits and appraise a higher sense of self-efficacy, which in turn achieves better work role performance. Thus, we propose the following:

Hypothesis 4a: Group-level LMX quality moderates the mediating effect of team member self-efficacy on the relationship between team leader autonomy-oriented help and individual work role performance of team members. Specifically, if the group-level LMX quality is higher, then the mediating effect of member self-efficacy is stronger.

Hypothesis 4b: Group-level LMX quality moderates the mediating effect of team member self-efficacy on the relationship between team leader dependency-oriented help and individual work role performance of team members. Specifically, if the group-level LMX quality is lower, then the mediating effect of member self-efficacy is stronger.

**Figure 1:**
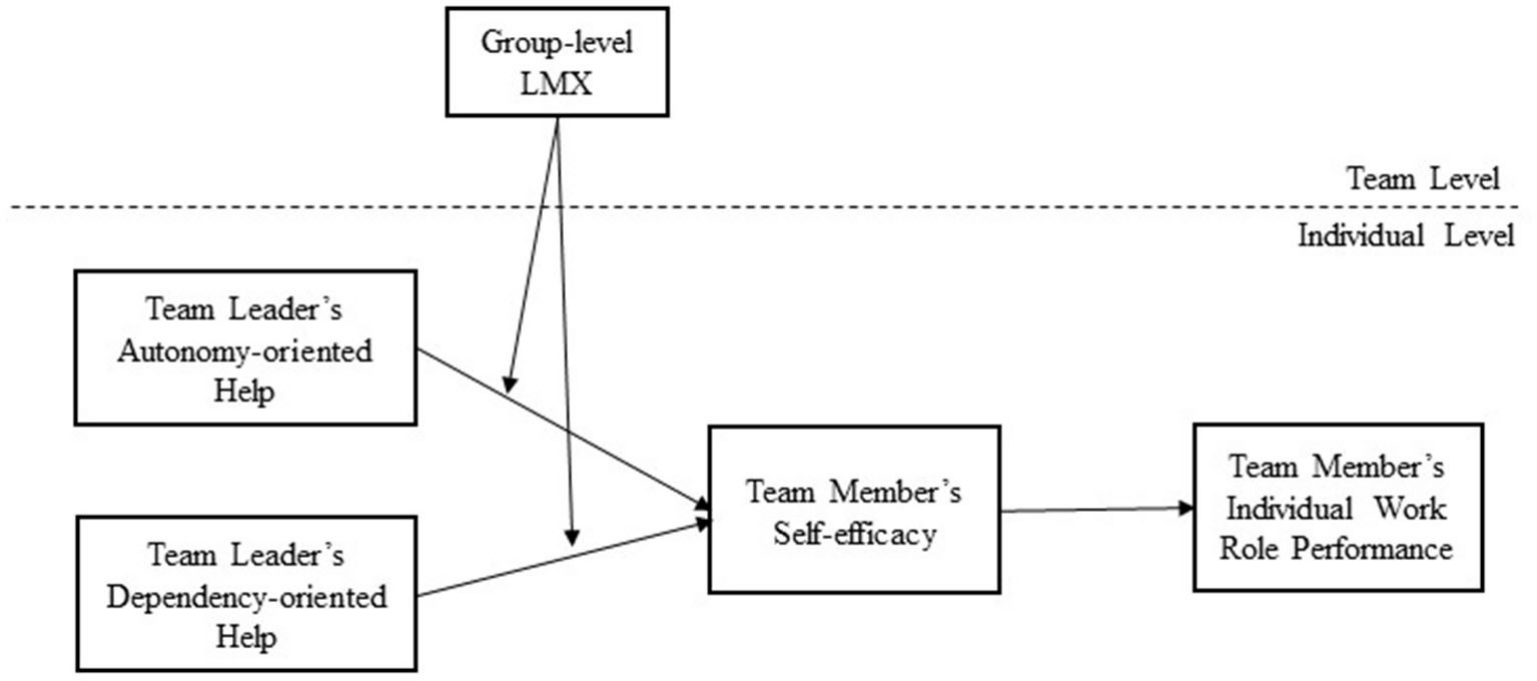
Hypothesized framework.

The hypothesized framework for this study is depicted in Figure 1.

## Scale Validation

Since there were few well-established scales for autonomy-oriented help and dependency-oriented help, we adopted a scale for each type of leader helping behaviors from the help orientation scale developed by Maki et al. (2017). A back-translation procedure (Brislin, 1986) was followed when using these two scales. A group of experienced people was invited to evaluate the content validity of these two scales, including six academic researchers in organizational behavior or management, eight team leaders or managers, and six front-line team members from different teams in different team-based organizations. First, we explained the definition and meaning of these two types of help to the group of people. With diverse experiences, this group of people understood and also agreed on the definitions of these two constructs from their personal perspectives. Then, each participant received a form containing 16 items (4 items of autonomy-oriented help, 3 items of dependency-oriented help, and 9 items of perceived supervisory support developed by Eisenberger et al., 2010) in a varying order to minimize the order effects (Hinkin and Tracey, 1999). Each form was provided with four categories (“autonomy-oriented help,” “dependency-oriented help,” “supervisory support,” and “unclassified”). Each participant was asked to assign these 16 items into groups as provided. The agreement indices for autonomy-oriented help and dependency-oriented help were satisfactory, which all of these participants can correctly assign scale items. It lent support for the content adequacy of these two measures (Hinkin, 1998). Second, we held a meeting with this group of people to discuss the item fit, item comprehension, and content clarity of autonomy-oriented help and dependency-oriented help scales. People within the group have consistent opinions on the fit, comprehension, and clarity of items for the scale of autonomy-oriented help. However, when evaluating the scale of dependency-oriented help, five leaders and three team members indicated that the item “My team leader helps me to meet my immediate work needs” needs further clarification in Chinese. Thus, we adjusted the Chinese wording of this item for the dependency-oriented help scale and kept the original content of the autonomy-oriented help scale based on the consensus reached in this meeting. Third, we followed the recommendation of Hinkin and Tracey (1999) to invite this group of people to rate the extent to which it is appropriate to measure both constructs (autonomy-oriented help and dependency-oriented help) on a five-point Likert scale from 1 (“not appropriate at all”) to 5 (“very appropriate”). In line with the definition of autonomy-oriented help, the ratings of content appropriateness on items of autonomy-oriented help (M = 4.43) are higher than those of dependency-oriented help (M = 1.48). As for the items of dependency-oriented help, content appropriateness scores were higher (M = 4.18) than those of autonomy-oriented help (M = 1.71). We conducted the analysis of variance on the difference between those two groups of scores for each scale, and both were significant (*p*'s < 0.001). In this way, the content of these two scales was validated.

Moreover, we conducted a pilot study for examining the scale validity by collecting data from an independent sample comprising 288 front-line employees from three middle-sized consulting companies in China (53% of the participants were male and 47% were female; 24% were between 20 and 30 years old, 42% were between 31 and 40 years old, and 34% were 41 years old and above). In this pilot study, the instructions of the survey explicitly informed the participants about research purpose and data confidentiality. The participants were requested to respond to the scale ranging from 1 = “strongly disagree” to 5 = “strongly agree” to assess the listed statements about the two types of help provided by their leaders. Cronbach's alpha was 0.72 for the autonomy-oriented help scale and 0.77 for the dependency-oriented help scale. The descriptive statistics and item analysis were shown in Table A1. To examine the discriminant validity of the adopted scale, we implemented confirmatory factor analysis for a two-factor model (autonomy-oriented help and dependency-oriented help) and a single-factor model (in which these two types of help were influenced by one latent factor). The results clarified that the two-factor model had a better model fit (χ^2^ = 27.20, *df* = 12, RMSEA = 0.07, SRMR = 0.05, CFI = 0.97, TLI = 0.95) than the single-factor model (χ^2^ = 167.01, *df* = 14, RMSEA = 0.20, SRMR = 0.11, CFI = 0.72, TLI = 0.57). These results support the reliability and validity of the autonomy-oriented help and dependency-oriented help scales in the Chinese context.

## Primary Study: Method

### Sample and Procedures

The sample for this study was composed of 39 work teams from five Chinese companies in China, including several industries of informational technology, telecommunications, finance and investment banking, and vehicle sales. We distributed questionnaires to 365 team members of 40 frontline work teams. In each company, an HR staff helped monitor the entire data collection process to ensure data quality. To minimize potential common method biases (Podsakoff et al., 2003), data were collected in three waves with a one-month interval between each wave on a time-lagged basis. The instructions on the questionnaire clarified that all information would be kept as confidential and that the questionnaire was anonymous. Additionally, general explanations and examples of autonomy-oriented help and dependency-oriented help were provided within the questionnaire in order to minimize potential confusion of the two constructs. The participants were requested to fill out their demographic information and rate the leader helping behaviors as well as LMX quality with their leaders at Time 1. Then, they rated their perceived self-efficacy at Time 2. We invited the corresponding team leaders to rate the individual work role performance of their members at Time 3. Each participant can receive an incentive (RMB 10 yuan) upon submitting their complete surveys. To further improve the validity of responses, an attention-check question was included in the survey.

We conducted data cleansing procedures suggested by Wen et al. (2018). For example, in the initial data filtering stage, surveys with inattentive and abnormal scores (e.g., all scores are basically the same) were eliminated. Since we obtained high commitment of top management teams of participating companies in this research, 331 team members returned their surveys at Time 1, with a response rate of 90.68%. At Time 2, we distributed surveys to these 331 team members and obtained 303 usable surveys, with a response rate of 91.54%. At Time 3, we distributed leader-version surveys to relative team leaders of 39 teams. All the corresponding team leaders who immediately supervised these 303 team members returned their surveys. We finally collected 303 valid sets of leader–member dyad survey data from 39 work teams, with an effective final response rate of 83.01%. Within the valid sample of team members, 156 participants (51.49%) were male and 147 participants (48.51%) were female; 174 participants (57.43%) were under 30 years old, 110 (36.30%) were between 31 and 40 years old, and 19 (6.27%) were between 41 and 50 years old; 32 participants (10.56%) had a degree of senior high school or below, 212 participants (69.97%) had a college diploma or bachelor's degree, and 59 participants (19.47%) had a master's degree or higher. In terms of job tenure in organizations, 71 participants (23.43%) had worked with their companies for less than a year, 185 participants (61.06%) worked for 1–5 years, 26 participants (8.58%) work for 6–10 years, and 21 participants (6.93%) worked for 10 years or longer. At the team level, each team has an average of 7.77 team members.

### Measures

All measures were presented to the participants with a five-point Likert rating scale ranging from 1 (“strongly disagree”) to 5 (“strongly agree”). The questionnaires were administered and data were collected in China. As the same practices in the pilot study, a back-translation process was leveraged based on the recommendations of Brislin (1986). Specifically, these items were first translated from English to Chinese by a management professor and then another management professor helped to translate them back to English. Further, a third management professor was invited to compare and verify the translated version in Chinese against the English version for translation accuracy.

Autonomy-oriented help. With four items, the autonomy-oriented help of team leaders was measured with the adapted scale of providing help orientation developed by Maki et al. (2017). The team members were asked to respond to items, such as “My team leader helps me develop the skill and knowledge to help myself.” Scale validity was examined using an independent sample in the pilot study. One item, “My team leader helps me to make sure I can eventually take care of my own needs,” was excluded because of its factor loading being not >0.50 in the confirmatory factor analysis. The alpha reliability in the main study was 0.90.

Dependency-oriented help. With three items, the dependency-oriented help of team leaders was measured with the adapted the scale of providing help orientation developed by Maki et al. (2017). The team members were asked to respond to items, such as “My team leader helps me by fixing problems for me.” Scale validity was examined in the pilot study as well. The alpha reliability in the main study was 0.86.

Individual self-efficacy. The individual self-efficacy of team members was measured with the eight-item self-efficacy scale developed by Chen et al. (2001). The scale has been tested with good reliability and validity in the Chinese context (e.g., Liao et al., 2010). An example of the items is “I am confident that I can perform effectively on many different tasks.” One item, “Even when things are tough, I can perform quite well,” was excluded because of its factor loading being not >0.50 in the confirmatory factor analysis. The alpha reliability for team member self-efficacy was 0.94.

Group-level LMX quality. Based on Graen and Uhl-Bien (1995), the LMX-7 is the most suitable scale for measuring the LMX as a unidimensional construct, and its reliability and validity were also empirically examined by Chinese scholars (e.g., Qu et al., 2013; Jiang and Yang, 2014). We measured the LMX quality perceived by team members with the seven-item LMX scale developed by Graen and Uhl-Bien (1995). The team members were invited to respond to items, such as “How well does your leader recognize your potential?” Two items measuring support and understanding were excluded because of their factor loadings being not >0.50 in the confirmatory factor analysis: “How satisfied your leader is with what you do?” “What are the changes that your leader would use his/her power to help you solve problems in your work?” For the five other remaining items, they are still representative to measure leader–member trust, understanding, support, and relationship quality. In this study, the alpha reliability for the LMX scale was 0.91. A one-way ANOVA was performed on LMX as a dependent variable and found that the between-group variance of LMX was significant (*F* = 3.43, *p* < 0.001). Since the team members are nested in different work teams, the within-group interrater reliability [R_wg(*j*)_] and intra-class correlation (ICC1 and ICC2) scores were calculated to identify whether the individual LMX ratings have between-group variance and whether such ratings can be aggregated to a team level. As the results displayed, the mean R_wg(*j*)_ of group-level LMX was 0.84 (>0.70), ICC1 = 0.24, ICC2 = 0.71, which suggested that the aggregation criterion was met (Klein et al., 2000). We hereby aggregated the individual LMX data to the team level in this research.

Individual work role performance. The individual work role performance of team members was measured with the nine-item individual work role performance scale developed by Griffin et al. (2007). The scores of individual work role performance were rated by the corresponding team leaders at Time 3 (two months after Time 1). Examples of items are “This team member carried out the core parts of his/her job well;” “This team member adapted well to changes in core tasks” and “This team member initiated better ways of doing his/her core tasks.” Consistent with other studies on work role performance, in this study, we used supervisor ratings of individual work role performance for potentially higher external validity (Hoffman et al., 1991). The alpha reliability for the individual work role performance of the team members was 0.95.

Control variables. Demographic information, such as gender, age, education levels, and organizational tenure of team members were measured as control variables, since they may exert potential impacts on individual self-efficacy and work performance in previous empirical studies (e.g., Seers, 1989; Stajkovic and Luthans, 1998; Erdogan and Liden, 2002; Liao et al., 2010; Liu et al., 2017; García-Chas et al., 2019). In addition, to differentiate the consequences between autonomy-oriented help and dependency-oriented help, the effect of dependency-oriented help was controlled for when testing the hypotheses regarding autonomy-oriented help; and vice versa. We executed all analyses including and excluding control variables (Becker, 2005). The results without control variables were consistent with those containing control variables.

Analytic strategy. First, we conducted analyses of descriptive statistics, correlations, and scale reliability in IBM SPSS 25.0. Second, since the data of the independent variables, mediator, and moderator in this study were self-reported, we evaluated the common method variance of this study by conducting a single unmeasured latent method factor test. Third, confirmatory factor analysis was implemented in Mplus 7.4 (Muthén and Muthén, 1998-2017) to assess the discriminant validity of this model. Fourth, as shown above, the ICC1 value (0.24) for group-level LMX indicates that the between-group variance was significant. Given the collected data of this study was a multilevel structure, we tested the hypotheses with multilevel modeling in Mplus 7.4.

## Results

### Results of Confirmatory Factor Analysis

Since the variables in this study were measured with scales, we conducted a confirmatory factor analysis (CFA) for discrimination validity test purpose. The CFA results indicated that a five-factor model [χ^2^ = 882.69, *df* = 311, root mean square error of approximation (RMSEA) = 0.06, standardized root mean square residual (SRMR) = 0.05, comparative fit index (CFI) = 0.93, non-normed fit index (NNFI) or (TLI) = 0.92] has a good model fit. Moreover, we tested a four-factor model by combining autonomy-oriented help and dependency-oriented help as one latent factor, but the results of the model fit indices were getting worse (χ^2^ = 1200.43, *df* = 315, RMSEA = 0.07, SRMR = 0.07, CFI = 0.88, TLI = 0.86). We also tested a single-factor model, and found that the fit indices were unacceptable (χ^2^ = 3168.88, *df* = 324, RMSEA = 0.13, SRMR = 0.11, CFI = 0.60, TLI = 0.56). Thus, compared with other alternative models, the hypothesized five-factor model contributed to a better model fit in this study based on the CFA results.

In addition, we performed a single unmeasured latent method factor test (Podsakoff et al., 2003) to evaluate whether there was a potentially serious common method variance. After adding a general method latent factor to the five-factor model, the model did not have significant improvement on data fit (χ^2^ = 878.83, *df* = 284, RMSEA = 0.07, CFI = 0.90, TLI = 0.87). Given the chi-square difference test is sensitive to the sample size when >200 (Cheung and Rensvold, 2002), it is appropriate to compare the change in TLI between competing models (Little, 1997). The TLI of the trait/method model was 0.87, which did not have improvement compared with that of the hypothesized five-factor model (TLI = 0.90). Thus, these CFA results of the discriminant validity and a single unmeasured latent method construct approach indicated that the common method variance was not serious in this study.

### Results of Descriptive Statistics and Correlations

The means, standard deviations, correlations, and reliability for the variables are presented in Table 1 below. The variables (e.g., autonomy-oriented help, dependency-oriented help, self-efficacy, LMX, and individual work role performance) were significantly correlated (*p* < 0.01), which provided the foundation of hypotheses testing in this study.

**Table 1:** Means, standard deviations, correlations, and reliability.

**Variables**	**Mean**	**Standard deviation**	**1**	**2**	**3**	**4**	**5**	**6**	**7**	**8**	**9**
1. Age^a^ (T1)	–	–	–								
2. Gender^b^ (T1)	–	–	0.06	–							
3. Education^c^ (T1)	–	–	0.06	0.16^**^	–						
4. Tenure^d^ (T1)	–	–	0.53^**^	0.12^*^	0.36^**^	–					
5. Dependency-oriented help (T1)	3.31	1.11	0.09	0.03	−0.16^**^	−0.03	(*0.86*)				
6. Autonomy-oriented help (T1)	3.97	0.95	0.10	−0.19^**^	−0.21^**^	−0.07	0.59^**^	(*0.90*)			
7. Self-efficacy (T2)	4.26	0.64	0.15^*^	−0.08	−0.08	0.02	0.32^**^	0.50^**^	(*0.94*)		
8. Work role performance (T3)	4.06	0.60	0.14^**^	−0.12^*^	−0.14^*^	−0.02	0.34^**^	0.40^**^	0.69^**^	(*0.95*)	
9. LMX (level 2; T1)	3.89	0.48	0.12^*^	−0.12^*^	−0.13^*^	−0.01	0.37^**^	0.53^**^	0.33^**^	0.29^**^	(*0.91*)

* *p < 0.05*,

** *p < 0.01 (two-tailed). N = 303 at the individual level (variables 1–8); N = 39 at the team level (variable 9). T1, T2, and T3 represent Times 1, 2, and 3, respectively*.

a *Age included four levels, which were (1) below 30 years old, (2) 31–40 years old, and (3) 41–50 years old, (4) 51 years old and above*.

b *Gender was coded as 0 (male) and 1 (female)*.

c *Education included three levels, which were (1) senior high school degree and below, (2) college diploma or bachelor's degree, and (3) masters' degree and above*.

d *Tenure included four levels, which were (1) below 1 year, (2) 1–5 years, (3) 6–10 years, and (4) above 10 years. The scale reliabilities are italicized as presented in the diagonals*.

### Hypotheses Testing

We leveraged the multilevel modeling to test the hypotheses of this study. With recommendations from Preacher et al. (2010), the Monte Carlo Method was adopted to validate the mediating and moderated mediation effects of this multilevel model and 95% confidence intervals were used to evaluate the effect significance. The results are presented in Tables 2, 3. First, as shown in Model 1 of Table 2, upon controlling for demographic variables, leader autonomy-oriented help was positively associated with member self-efficacy (γ = 0.30, *p* < 0.001), while the effect of leader dependency-oriented help on member self-efficacy was marginally significant (γ = 0.06, *p* = 0.08). Thus, H1 was supported. Second, as shown in Model 3, autonomy-oriented help was positively correlated with individual work role performance (γ = 0.16, *p* < 0.001) when the effect of dependency-oriented help was considered (γ = 0.12, *p* < 0.01). Third, when including both autonomy-oriented help and self-efficacy into Model 4, the self-efficacy of members became positively associated with their individual work role performance (γ = 0.64, *p* < 0.001) when the direct effect of autonomy-oriented on work role performance was controlled for and, in turn, became insignificant (γ = −0.05, *n.s*.). It provided support for the mediating role of self-efficacy in the relationship between autonomy-oriented help and work role performance. Further, the results of the Monte Carlo method (20,000 replications) in Table 3 show that the indirect effect of autonomy-oriented help on work role performance through self-efficacy was significant and positive, and the 95% confidence interval (CI) did not include zero (indirect effect = 0.21, CI = [0.11, 0.32]). Thus, member self-efficacy mediated the relationship between leader autonomy-oriented help and the individual work role performance of members. H2a was hereby supported. On the other hand, given there was a positive relationship between dependency-oriented help and work role performance, we continued to examine the indirect effect between each other (Wen and Ye, 2014). As Table 3 indicated, the Monte Carlo method (20,000 replications) indicated that the 95% CI included zero (indirect effect = 0.04, CI = [−0.005, 0.10]), which means that the indirect effect of self-efficacy between dependency-oriented help and individual work role performance is insignificant and H2b was not supported.

**Table 2:** Unstandardized coefficients of the hypothesized model.

**Variables**	**Self-efficacy**		**Work role performance**	
	**(Level 1; T2)**		**(Level 1; T3)**	
	**Model 1**	**Model 2**	**Model 3**	**Model 4**
**Control variables (level 1)**				
Gender	−0.001	−0.04	−0.09	−0.09
Age	0.12	0.06	0.12^**^	0.06
Education	0.07	0.01	−0.03	−0.08
Tenure	−0.01	0.04	−0.04	−0.03
**Independent variable (level 1)**				
A-Help^a^ (T1)	0.30^***^	0.41^***^	0.16^***^	−0.05
D-Help^b^ (T1)	0.06^†^	0.01	0.12^**^	0.09^**^
**Mediator (level 1)**				
Self-efficacy (T2)				0.64^***^
**Moderator (level 2)**				
LMX (T1)		0.45^***^		
**Cross-level interactions**				
A-Help × LMX		0.22^***^		
D-Help × LMX		0.14^†^		

† *p < 0.1*,

** *p < 0.01*,

*** *p < 0.001 (two-tailed). N = 303*.

a *Autonomy-oriented help*;

b *Dependency-oriented help. T1, T2, T3 represent Time 1, 2, and 3, respectively*.

**Table 3:** Indirect effects of leader helping on member work role performance.

**Effect**	**Mediation path**	**Estimate**	**SE**	**95% CI**
Indirect effect	Autonomy-oriented help → Self-efficacy → Work role performance	0.21^***^	0.06	0.11	0.32
	Dependency-oriented help → Self-efficacy → Work role performance	0.04^†^	0.02	−0.005	0.10

† *p < 0.1*,

*** *p < 0.001 (two-tailed). N = 303*.

**Figure 2:**
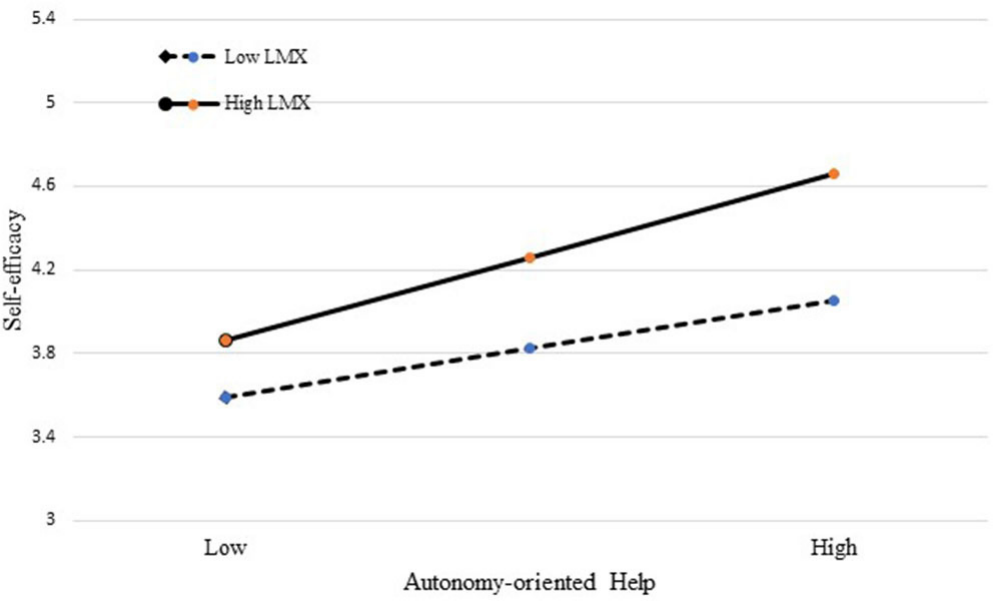
The interactive effect of autonomy-oriented help and group-level LMX on self-efficacy.

Instructed by Hofmann and Gavin (1998), the data of autonomy-oriented help and dependency-oriented help (as the independent variables) were group-mean centered, and the data of group-level LMX (as the moderator) were grand-mean centered. The results of Model 4 indicated that the interaction of autonomy-oriented help and group-level LMX was positively and significantly related to self-efficacy (γ = 0.22, *p* < 0.001). Thus, H3a was supported. Further, we conducted a simple slope test to better illustrate the interactive effect of autonomy-oriented help and group-level LMX on self-efficacy (see Figure 2). Specifically, the relationship between autonomy-oriented help and self-efficacy was stronger with high group-level LMX; while such relationship was weaker with low group-level LMX. However, the interaction of dependency-oriented help and group-level LMX was insignificantly related to self-efficacy (γ = 0.14, *p* = 0.06). Thus, H3b was not supported.

In order to further explore the effects of the mediating role of member self-efficacy on the relationship between the autonomy-oriented help of team leaders and the individual work role performance of team members, we integrated and tested the moderated mediating effect of this model by utilizing the analytical techniques recommended by Edwards and Lambert (2007). The Monte Carlo method (20,000 replications) was utilized. The results are shown in Table 4 below. As illustrated, the indirect effect of autonomy-oriented help on individual work role performance was significant with high group-level LMX, and the 95% CI was zero excluded (CI = [0.18, 0.38]); while such indirect effect was significant with low LMX (CI = [0.07, 0.25]; zero excluded). Further, the indirect effect was significant at the level of LMX difference (CI = [0.01, 0.21]). Thus, H4a was supported. However, H4b was not supported. Specifically, the indirect effect of self-efficacy in the relationship between dependency-oriented help and work role performance was significant with high group-level LMX (95% CI = [0.02, 0.13]), but insignificant at both levels of low LMX (95% CI = [−0.08, 0.06]) and LMX difference (95% CI = [−0.003, 0.17]).

**Table 4:** Moderated mediating effect of self-efficacy on the relationship between autonomy-oriented help and work role performance at different LMX levels.

**Group-level LMX**	**Indirect effect**	**S.E**.	**95% CI**	
High	0.28^***^	0.05	0.18	0.38
Low	0.16^***^	0.05	0.07	0.25
Difference	0.11^**^	0.05	0.01	0.21

** *p < 0.01*,

*** *p < 0.001 (two-tailed). N = 303*.

### Supplementary Analyses

**Figure 3:**
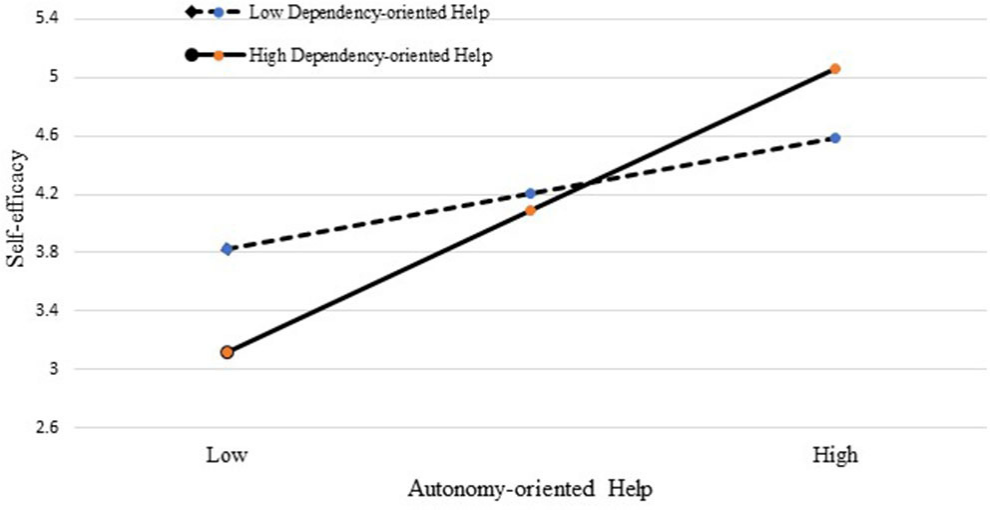
The interactive effect of autonomy-oriented help and dependency-oriented help on self-efficacy.

In order to further explore whether the relationship between autonomy-oriented help and dependency-oriented help are synergistic or compensatory, we also examined the interaction effect between these two types of helping on individual self-efficacy and in turn work role performance at the individual level. The supplementary analyses revealed that the interaction of autonomy-oriented help and dependency-oriented help was positively and significantly related to self-efficacy (γ = 0.28, *p* < 0.001). Accordingly, Figure 3 and the simple slope test indicated the moderating role of dependency-oriented help in the relationship between autonomy-oriented help and self-efficacy, and the indirect effect of autonomy-oriented help on individual work role performance *via* self-efficacy was significant at a high level of dependency-oriented help and the bias-corrected bootstrapping CI was zero excluded (95% CI = [0.49, 0.94]); while such indirect effect was also significant at a low level of dependency-oriented help (95% CI = [0.19, 0.48]; zero excluded). Further, the indirect effect was significant at the level of dependency-oriented help difference (95% CI = [0.16, 0.55]). Therefore, it is interesting and of academic value to find the relationship between the two types of help of leaders is not contrasting but synergistic when forming self-efficacy of employees, in turn, influencing their work role performance.

## Discussion

Based on the social cognitive theory (Bandura, 1997), this study investigates the mechanism of individual self-efficacy between the help of team leaders (in terms of autonomy-oriented help and dependency-oriented help) and the individual work role performance of team members. The research also explored the moderating role of group-level LMX and the moderated mediating effect at different LMX quality levels through data analysis on 303 valid responses. The results indicate, first, that these two types of leader helping exert different influences on member self-efficacy. Specifically, the autonomy-oriented help of team leaders is positively and significantly related to the self-efficacy of team members, while dependency-oriented help of leaders does not significantly contribute to individual self-efficacy. The explanation for such findings is that autonomy-oriented help, a form of help representing educational value (e.g., Nadler, 1997), is more likely to foster higher self-efficacy of team members compared with offering them dependency-oriented help. This is consistent with the findings of prior research. Dependency-oriented help of leaders can serve as instant input on the work role performance of members but fails to sufficiently empower them. This also can explain why there is a positive relationship between dependency-oriented help and work role performance other than self-efficacy in this study. Interestingly, it brings new insights to the dependency-oriented help literature by suggesting a bright side for team members when receiving dependency-oriented help, especially for newcomers who confront the uncertainty and variability of the organizational environment.

Second, the indirect effect of member self-efficacy between the autonomy-oriented help of team leaders and member work role performance is significantly stronger than that for dependency-oriented help of leaders. To be more specific, compared with dependency-oriented help, the autonomy-oriented help of leaders can engender a differential positive effect on the self-efficacy of members toward their individual work role performance through their cognitive, motivational, and selection processes (Bandura, 1994). However, the effect of the dependency-oriented help of leaders on the work role performance of members is not mediated by the self-efficacy of members, which implies that alternative mechanisms may exist. That occurs because employees cognitively use many cues from different perspectives (e.g., internal cues, such as personal abilities, efforts, luck, and motivation; external cues, such as task characteristics, task resources, and group interdependence) to analyze and form efficacy self-appraisals (Gist and Mitchell, 1992). For example, an individual routinely judges their self-efficacy with increasing experiences on tasks; otherwise, they may consider “the task demands,” situational constraints, available resources, personal attributes, and feelings to assess their self-efficacy when meeting a new or challenging task (Gist and Mitchell, 1992, p. 191). In other words, it is possible that employees evaluate other social cues and job factors to assess their self-efficacy when perceiving the dependency-oriented help of leaders, especially in a demanding situation. Moreover, the employees who participated in this study are highly educated and work in industries that are knowledge-intensive. The initial level of self-efficacy of survey respondents may be relatively high and stable. In this way, the dependency-oriented help of leaders may not be a primary factor to influence the self-efficacy of members or, in turn, their work role performance.

Third, the group-level LMX quality can positively moderate the effect of autonomy-oriented help and self-efficacy on individual work role performance, but not that of dependency-oriented help. One explanation of these results may be the cultural factors of LMX exist in this study (Rockstuhl et al., 2012). In the Chinese context, team members are more likely to work hard for their leaders even if they do not receive adequate or expected resources from their leader (Chen et al., 2009). Thus, when receiving dependency-oriented help of leaders, which may be of less long-term educational value, team members tend to proactively secure other types of job resources to obtain a certain level of self-efficacy for work role performance regardless of the level of group-level LMX in the team. In contrast, team leaders high in LMX are more likely to act similarly by providing their members with abundant resources, leader support, and important tasks across different cultures (Rockstuhl et al., 2012). Team members across cultures similarly need and cherish these resources for good work performance (Rockstuhl et al., 2012). Thus, the self-efficacy of team members can be further enhanced by leveraging the autonomy-oriented help of their leaders in conjunction with other job resources from leaders and colleagues in a positive team atmosphere from high group-level LMX. In turn, team members are more likely to feel capable and motivated to take actions in terms of task proficiency, adaptivity, and proactivity to achieve outstanding individual work role performance. Therefore, the indirect effect of self-efficacy on the relationship between the autonomy-oriented help of leaders and the self-efficacy of members is contingent on the factor of group LMX quality.

Finally, it is worth noting that the synergistic (rather than compensatory) interaction of the autonomy-oriented help and dependency-oriented help of leaders can influence the self-efficacy of members and further their work role performance at the individual level. Inspired by the supplemental analyses in this study, autonomy-oriented help can synergistically work with dependency-oriented help on individual work role performance *via* self-efficacy. The reason may be that both types of leader helping provide members with sufficient support of instrumentality and sustainability, which fuels great power to member work performance through their self-efficacy enhancement.

## Implications and Conclusions

This study contributes to the literature in several ways. First, this study integrates and contributes to the organizational citizenship behavior (OCB) and helping literature. Helping was put as a simple act in prior OCB literature and few researchers conducted studies on different types of help as well as their differential mechanisms. Ehrhart (2018) appealed that more academic attention should be paid across “various types of help and levels of analysis” of the helping process (p. 1). In addition, most studies on autonomy/dependency-oriented help have mainly focused on psychological consequences in individuals rather than behavioral outcomes. By exploring the deeper meaning of helping (a critical form of OCB), this study illustrates the indirect impacts of the autonomy-oriented help and dependency-oriented help of team leaders on the work performance of members *via* the psychological mechanism of personal self-efficacy and the boundary condition. It theoretically and empirically enriches research findings on different types of help and responds to the appeal of exploring the “how, when, and why” of the helping process, as described by Ehrhart (2018).

Second, drawing upon the social cognitive theory, this study investigates different indirect effects of leader helping behaviors on individual work role performance of members through their self-efficacy. The integrative framework of two different types of help provides an interesting perspective to understand further that helping is not just about “lift and shift” work (dependency-oriented help) but also about transforming work (autonomy-oriented help). The research finding on receiving autonomy-oriented help is consistent with prior studies. However, it is encouraging that dependency-oriented help can play a positive role in promoting individual work role performance. Compared with prior studies with emphases on negative impact, this study empirically investigates the bright side of dependency-oriented help for the help recipient. It also suggests that these two types of help can function together on perceptions and behaviors of team members to a certain degree.

Third, few literature has uncovered the boundary conditions of autonomy-oriented help. This research provides insight on the social contextual factor of LMX and demonstrates the moderating effect of group-level LMX and the moderated mediating effect of self-efficacy at various LMX levels; which stresses a deeper understanding of the consequences of leader helping behaviors in a cross-level setting. Fourth, this study improves the understandings of the work role performance. It addresses the dispositional and contextual factors of work role performance at the individual level by elaborating on “mechanisms through which the characteristics of people and situations influence behaviors within the specific subdimensions of the model” (Griffin et al., 2007, p. 343). In addition, this study presents the cognitive and motivational working process of leader helping with the self-efficacy perceptions of members. This makes a critical contribution to the literature of self-efficacy and responds to the appeal of systematically and empirically understanding different effects of determinants of self-efficacy (Gist and Mitchell, 1992).

Further, this study also highlights several practical implications: (1) helping can be leveraged as a management strategy to improve leadership effectiveness at the individual level. This study implies that in organizations it may not be a one-fit-all solution for managers to “teach people how to fish” instead of simply giving them fish. Managers should leverage the differential effects of different types of help for their team members. They may have theoretical references for learning when and how to provide a fish (dependency-oriented help) or a fishing tool (autonomy-oriented help). Although providing a fish cannot significantly enhance personal self-efficacy, managers are not necessary to teach members how to fish in all circumstances. Giving someone a fish can sometimes turn to be beneficial to their work performance given the task attributes and situational constraints. When choosing whether to offer dependency-oriented help or autonomy-oriented help, a team leader also needs to consider the contextual factor, such as group-level LMX. Furthermore, a team leader should balance the short-term and long-term effects of dependency-oriented help. Although the performance of team members can be enhanced because of the dependency-oriented help of leaders in a short run, it may make members become highly dependent on the long-term help of leaders, which is possibly detrimental for both leaders and members. (2) Aside from the quality of exchange relationship in a team, managers may need to account for task attributes and characteristics. For novel but not urgent work tasks, it would be a good training opportunity to provide autonomy-oriented helping as an aid in long-term sustainability, since employees would leverage personal and situational resources to enhance their self-efficacy and, in turn, explore solutions, especially in a high LMX team climate. For interdependent or time-limited work tasks, it is acceptable for leaders to use a dependency-oriented helping strategy to meet work demands, which can at least instantly benefit member work performance. Sometimes it could be effective to apply a dependency-oriented helping strategy to newcomers to facilitate their socialization and adaptation processes within a team. However, as the employee work experiences increase, managers should leverage the art of the combination of both types of helping to maximize their positive impacts on the work role performance of subordinates through lifting their self-efficacious belief level. (3) Further, team leaders and managers can enhance the general LMX quality of their teams by providing more communication, autonomy, empowerment, and other supportive resources to members. (4) Employees (or as team members) need to cherish the value of autonomy-oriented help and should not underestimate the value of dependency-oriented help toward their work performance, since receiving either type of help of leaders can generate distinguishable influences. Employees can also proactively ask for autonomy-oriented help from their supervisors if the situation is not urgent.

This study has some limitations. First, the data collected are cross-sectional because of practical constraints. Although we conducted the time-lagged measurement method (Podsakoff et al., 2003), future research can implement a longitudinal research design with multiple sources and multiple studies of data collection to minimize potential common method bias. Second, future studies should also consider the time horizon into the research design. Since the bright side of the dependency-oriented help of leaders is instantly helpful and instrumental to employee performance, it is necessary to study the long-term effects of dependency-oriented help on individual work performance across time. Third, the Chinese employees “respect the authority associated with hierarchical positions” (Chen et al., 2014, p. 812), given the nature of Chinese society. Receiving help from leaders has a much greater impact than that from other sources (e.g., co-workers) in the Chinese context. Thus, we did not measure the help of coworkers, although it may be an alternative predictor of the self-efficacy of an individual. However, future research may include peer support as a control variable to see whether there are interesting findings. Fourth, individual differences may exert potential moderating roles, since some personal traits (e.g., the influence of self-esteem on self-efficacy, and openness to change as a factor of work role performance) were examined to have an impact on perceived self-efficacy (Brockner, 1979) and work role performance (Griffin et al., 2007). Future researchers should consider incorporating dispositional variables into the framework for exploring other possible moderating roles of the relationship between the help of leaders and the self-efficacy of subordinates. Finally, the supplementary analyses found that the dependency-oriented help can play a positive moderating role in the indirect path between autonomy-oriented help and work performance. The reason may be that help of instrumentality and information can serve as instant resource input for knowledge-intensive workers especially in the challenging era of today. Furthermore, this study was conducted in China. The generalizability of the conclusions may be limited in other countries. Future researchers can collect data from different countries or different cultures to obtain more interesting cross-cultural insights.

## Data Availability Statement

The raw data supporting the conclusions of this article will be made available by the authors, without undue reservation.

## Ethics Statement

Ethical review and approval was not required for the study on human participants in accordance with the local legislation and institutional requirements. The patients/participants provided their written informed consent to participate in this study.

## Author Contributions

YZ developed the theoretical model and wrote the manuscript, with the contribution of LZ. YZ, LZ, and YG reviewed the literature. LZ revised the manuscript. YG participated in the overall discussion on the project and manuscript revision. All authors contributed to the article and approved the submitted version.

## Conflict of Interest

The authors declare that the research was conducted in the absence of any commercial or financial relationships that could be construed as a potential conflict of interest.

## Publisher's Note

All claims expressed in this article are solely those of the authors and do not necessarily represent those of their affiliated organizations, or those of the publisher, the editors and the reviewers. Any product that may be evaluated in this article, or claim that may be made by its manufacturer, is not guaranteed or endorsed by the publisher.

## Appendix

**Table A1:** Analysis of autonomy-/dependency-oriented help scale items.

**Items**	**M**	**SD**	**Corrected item-total correlation**	**Factor loading**
**Autonomy-oriented Help (Cronbach's** **α: 0.72)**				
1. My team leader helps me to make sure I can eventually take care of my own needs.	3.90	0.91	0.43	0.52
2. My team leader helps me improve my abilities to fix my own problems.	4.08	0.84	0.58	0.83
3. My team leader helps me learn how to solve my own problems.	4.13	0.85	0.54	0.75
4. My team leader helps me develop the skill and knowledge to help myself.	4.19	0.83	0.47	0.74
**Dependency-oriented help (Cronbach's** **α: 0.77)**				
1. My team leader gets involved in taking care of my problem.	3.01	1.13	0.69	0.86
2. My team leader helps me by fixing problems for me.	3.27	1.17	0.65	0.83
3. My team leader helps me to meet my immediate work needs.	3.32	1.14	0.60	0.78

*M, mean; SD, standard deviation. N = 288*.
